# MRI recovery with self-calibrated denoisers without fully-sampled data

**DOI:** 10.1007/s10334-024-01207-1

**Published:** 2024-10-16

**Authors:** Muhammad Shafique, Sizhuo Liu, Philip Schniter, Rizwan Ahmad

**Affiliations:** 1 Biomedical Engineering, Ohio State University, Columbus, OH 43210 USA; 2 Electrical and Computer Engineering, COMSATS University, Islamabad, Pakistan; 3 Electrical and Computer Engineering, Ohio State University, Columbus, OH 43210 USA

**Keywords:** Self-supervised, MRI, Reconstruction

## Abstract

**Objective:**

Acquiring fully sampled training data is challenging for many MRI applications. We present a self-supervised image reconstruction method, termed ReSiDe, capable of recovering images solely from undersampled data.

**Materials and methods:**

ReSiDe is inspired by plug-and-play (PnP) methods, but unlike traditional PnP approaches that utilize pre-trained denoisers, ReSiDe iteratively trains the denoiser on the image or images that are being reconstructed. We introduce two variations of our method: ReSiDe-S and ReSiDe-M. ReSiDe-S is scan-specific and works with a single set of undersampled measurements, while ReSiDe-M operates on multiple sets of undersampled measurements and provides faster inference. Studies I, II, and III compare ReSiDe-S and ReSiDe-M against other self-supervised or unsupervised methods using data from T1- and T2-weighted brain MRI, MRXCAT digital perfusion phantom, and first-pass cardiac perfusion, respectively.

**Results:**

ReSiDe-S and ReSiDe-M outperform other methods in terms of peak signal-to-noise ratio and structural similarity index measure for Studies I and II, and in terms of expert scoring for Study III.

**Discussion:**

We present a self-supervised image reconstruction method and validate it in both static and dynamic MRI applications. These developments can benefit MRI applications where the availability of fully sampled training data is limited.

## Introduction

Magnetic resonance imaging (MRI) is a well-established diagnostic tool that offers several advantages over other imaging modalities, including excellent soft-tissue contrast, high spatial and temporal resolution, multiple contrast mechanisms, and radiation-free acquisition. MRI is routinely used in various clinical applications, including neuro, musculoskeletal, abdominal, and cardiovascular imaging. However, long scan times remain a challenge in MRI, as they can reduce patient comfort, increase sensitivity to motion, and decrease patient throughput. Consequently, accelerating MRI has become a highly active area of research [1].

In parallel MRI (pMRI), which is available on all commercial MRI scanners, data are acquired simultaneously across multiple receive coils [2]. Typically, pMRI can speed up the acquisition process by a factor of two to three for 2D planar imaging, while higher acceleration factors are achievable in multi-band and 3D imaging. To achieve further acceleration, pMRI can be combined with methods that utilize prior information about the image. For instance, compressed sensing (CS) leverages sparsity-based priors and can be effectively paired with pMRI [3]. The combination of pMRI and CS achieves higher acceleration rates than pMRI alone, and these reconstruction methods are increasingly available on commercial scanners [4]. More recently, deep learning (DL) methods have been developed to reconstruct images from highly undersampled MRI data. Several studies indicate that DL methods can outperform sparsity-driven compressed sensing (CS) methods in terms of image quality [5, 6]. In particular, end-to-end variational networks (VarNet), as early DL-based extensions of compressed sensing, have shown strong performance across various MRI reconstruction tasks and are considered a competitive benchmark in the field [7]. Most DL methods, including VarNet, are based on supervised learning, where a reconstruction network is trained on a large, fully sampled training dataset [8]. Outside of a few 2D applications, such training datasets are often unavailable. For other applications, such as cardiac imaging, collecting full resolution fully sampled data may not be feasible [9]. Consequently, self-supervised DL (SSDL) techniques have recently gained significant interest for MRI reconstruction [10]. These techniques do not require fully sampled training datasets and instead utilize the redundant information within the undersampled data to guide the training process.

Several SSDL methods have been proposed for image denoising, including single-instance deep generative prior methods such as deep image prior (DIP) and deep decoder [11, 12]. These methods model an image as the output of a generator network, with both network parameters and input code vectors trained on an image-specific basis. Another popular SSDL denoising method is Noise2Noise [13], which denoises images using two noisy copies of the same image. However, acquiring multiple copies of an image is not practical for most MRI applications. To address this issue, other SSDL-based denoising methods have recently been proposed, including Noise2Void [14], Noise2Self [15], and Self2Self [16], which operate on a single noisy image. These methods train a network to predict a pixel from its neighboring pixels or predict one group of pixels from another. Since noise is assumed to be independent across pixels, the network denoises the image by implicitly learning the underlying structure in the image. For SSDL-based denoising, Xu et al. took a different approach and proposed a method called Noisy-As-Clean [17]. This method works by adding synthetic noise to the noisy images and training a denoising network to remove the added noise. The trained network is then used to denoise the images on which it was trained. In a separate development, Stein’s unbiased risk estimator (SURE)-based loss was used for unsupervised training of denoising networks [18].

The application of SSDL extends beyond image denoising, and many of these methods can be applied to solve inverse problems, including image reconstruction. For instance, DIP can readily solve inverse problems with a known forward operator. Yoo et al. employed DIP for reconstructing dynamic MRI [19]. More recently, Bell et al. introduced a more robust adaptation of DIP by training the network to function as a denoiser instead of a generator [20]. In a separate study, Hamilton et al. integrated low-rank constraint with DIP and applied it to free-breathing cardiac imaging [21]. Scan-specific robust artificial neural network for k-space interpolation (RAKI) is another self-supervised method proposed for MRI reconstruction [22]. RAKI is similar to Noise2Void but operates in k-space. In RAKI, a network is first trained on the fully sampled auto-calibration signal (ACS) region and then used to predict missing k-space samples from neighboring measured samples. Both RAKI and its recent extension, called residual RAKI [23], can be viewed as nonlinear extensions of traditional GRAPPA [24]. However, due to their scan-specific nature, DIP and RAKI are computationally slow, which limits their translational potential. In 2020, Yaman et al. proposed self-supervised learning via data undersampled (SSDU), a self-supervised learning method that resembles Noise2Self, but with a loss function defined in k-space [25]. In SSDU, the acquired undersampled k-space is divided into two subsets, and an unrolled network is trained to infer images from the first subset such that those images are consistent with the second subset. At the inference stage, the trained network in SSDU can rapidly map an undersampled, aliased image to a fully sampled image. More recently, Millard and Chiew used the Noisier2Noise [26] framework to further improve SSDU by ensuring that the sampling masks of the two subsets have similar distributions [27]. Cole et al. [28] also proposed training a network to map undersampled, aliased images to fully sampled images but with an adversarial loss, where the discriminator is fed two unrelated undersampled images: one from the image reconstruction network output and one from an independent set of measurements. Finally, generalized SURE (GSURE), which extends SURE to inverse problems, has been proposed [29]. However, for practical inverse problems, the projected mean squared error used in GSURE often poorly approximates the true mean squared error. Aggarwal et al. [30] addressed this issue by using an ensemble approach, but it requires a collection of data undersampled with different sampling patterns, which may not be available. In another application of SURE for inverse problems [31], Zhussip et al. used SURE and an approximate message passing algorithm to train the denoiser from corrupted images for Gaussian measurement matrices. However, this method relies on the residual at each iteration of image reconstruction being approximately Gaussian, which is not typically the case for non-trivial forward operators.

In this work, we propose an SSDL method, called recovery with a self-calibrated denoiser (ReSiDe), for image reconstruction. Our main contribution involves integrating the self-supervised denoising scheme of Noisy-As-Clean [17] with the PnP framework to solve the inverse problem in MRI reconstruction. Additionally, we employ the discrepancy principle to iteratively adapt the strength of the denoiser, providing faster convergence and more control over the quality of the final reconstruction. Finally, we propose a faster, more robust version of ReSiDe that is applicable to cases where multiple undersampled sets of measurements are available. Using data from brain MRI, MRXCAT perfusion phantom, and first-pass perfusion MRI, we show that ReSiDe outperforms other self-supervised and unsupervised image reconstruction methods. These developments significantly expand our preliminary description of ReSiDe [32], which did not include auto-tuning, was applicable only to a single set of measurements, and used only one T1 and one T2-weighted image for validation.

## Materials and methods

### Plug-and-play based recovery

The MRI reconstruction entails estimating the underlying image from noisy and potentially undersampled k-space measurements. The measured noisy k-space data are related to the image via

1 $$\begin{aligned} \varvec{y} = \varvec{A}\varvec{x} + \varvec{\eta }, \end{aligned}$$

where $\varvec{x}\in {\mathbb {C}}^N$ is a vectorized *N*-pixel image, $\varvec{y}\in {\mathbb {C}}^M$ is the pre-whitened and scaled pMRI data from *C* receive coils, $\varvec{\eta }\in {\mathbb {C}}^M$ is circularly symmetric zero-mean white Gaussian noise with known variance $\sigma ^2$ that depends on the data scaling, and $\varvec{A}\in {\mathbb {C}}^{M\times N}$ is a known forward operator that subsumes coil sensitivity maps, discrete Fourier transform, and k-space undersampling.

To reduce acquisition time, the k-space data are often prospectively undersampled to achieve an acceleration rate $R\triangleq \frac{CN}{M} > 1$. At high acceleration rates, the problem in Eq. 1 becomes ill-posed. In that case, a common remedy is to inject prior knowledge about $\varvec{x}$ using a regularizer, resulting in the optimization problem of the form

2 $$\begin{aligned} \hat{\varvec{x}} = \mathop {\mathrm {arg\,min}}\limits _{\varvec{x}} \frac{1}{\sigma ^2}\Vert \varvec{y} - \varvec{A}\varvec{x} \Vert ^2_2 + \mathcal {R}(\varvec{x}), \end{aligned}$$

where the first term enforces data consistency and $\mathcal {R}(\cdot )$ represents the regularizer. For CS-based MRI reconstruction, popular choices for $\mathcal {R}(\varvec{x})$ include total variation [33] and $\lambda \Vert \varvec{\Phi } \varvec{x}\Vert _1$, where $\varvec{\Phi }$ represents a linear sparsifying transfrom, and $\lambda > 0$ controls the regularization strength [3]. It has been shown that a simple sparsity-based regularizer may not fully capture the rich structure in medical images [34]. To leverage more complex priors, Venkatakrishnan et al. proposed an algorithmic framework called plug-and-play (PnP) [35]. In PnP, an off-the-shelf image denoiser, $\varvec{f}(\cdot )$, is called within an iterative algorithm, e.g., primal-dual splitting (PDS) [36]. A PDS-based implementation of PnP is given in Algorithm 1. In the subsequent sections, we will use this algorithm as a starting point to explain ReSiDe. Note, the implementation of PnP or ReSiDe is not limited to PDS and can be carried out using other algorithms [37].

**Algorithm 1:**
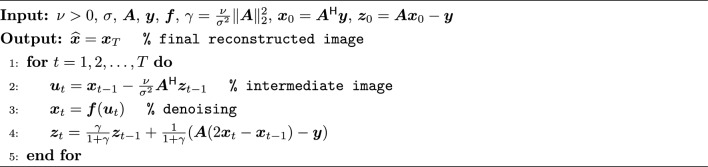
PnP implemented using PDS

### ReSiDe-S

ReSiDe-S is a scan-specific technique that operates on a single set of undersampled measurements, $\varvec{y}$. A PDS-based implementation of ReSiDe-S is given in Algorithm 2. ReSiDe-S differs from PnP in the way the denoiser $\varvec{f}(\cdot )$ is trained. The denoiser used in PnP is either generic or pre-trained using an additional training dataset. In contrast, the denoiser in ReSiDe-S is progressively trained from the image being recovered. This is akin to blind compressed sensing [38], where both the image and the sparsifying transform are simultaneously learned during the reconstruction process. Following the work by Xu et al. [17], we propose training a DL-based denoiser by adding synthetic noise to the image being recovered. The denoiser training process is described in Line 3 of Algorithm 2. In summary, $\varvec{u}_t$ is an intermediate image at iteration *t*, and $\mathcal {I}_i[\varvec{u}_t]$ represents the $i^\text {th}$ patch from $\varvec{u}_t$. For training purposes, $\mathcal {I}_i[\varvec{u}_t]$ and $\mathcal {I}_i[\varvec{u}_t] + \mathcal {N}(\varvec{0}, s_{t-1}^2 \varvec{I})$ act as “clean” and “noisy” patches, respectively. Here, $\mathcal {N}(\varvec{0}, s_{t-1}^2 \varvec{I})$ represents complex-valued zero-mean white Gaussian noise with variance $s_{t-1}^2$. The denoiser $\varvec{f}(\cdot ; \varvec{\theta })$, parameterized by $\varvec{\theta }$, is trained using $P\ge 1$ patches in a supervised fashion by minimizing the loss $\mathcal {L}(\cdot , \cdot )$, which measures the difference between the denoiser output and clean patches. Once the denoiser is trained, it is then used to denoise the intermediate image $\varvec{u}_t$ (Line 4 in Algorithm 2), but this time without the added noise. The process of training and applying the denoiser is repeated in each of *T* iterations or until some convergence criterion is reached.

The strength of the denoiser is controlled by $s_{t}^2$, which plays a role similar to that of regularization strength in CS, i.e., larger $s_{t}^2$ leads to smoother images and smaller $s_{t}^2$ leads to noisier but sharper images. As evident from our preliminary results [32], the value of $s_{t}^2$ also controls the speed of convergence, with larger values preferred in the earlier iterations to speed up convergence and smaller values preferred in later iterations to avoid over-smoothing of the recovered images. To adapt the value of $s_{t}^2$ over iterations, we propose using Morozov’s discrepancy principle [39, 40] (Line 6 and Line 7 in Algorithm 2). The discrepancy principle is based on the assumption that the squared residual norm, $\Vert \varvec{A}\varvec{x}_t - \varvec{y}\Vert _2^2$, monotonically increases with $s_{t}^2$, i.e., more aggressive denoising takes the image away from the least squares solution. By exploiting this monotonic relationship, the discrepancy principle updates $s_{t}^2$ such that the value of $\Vert \varvec{A}\varvec{x}_t - \varvec{y}\Vert _2^2$ approaches $M\sigma ^2$, which is the expected value of the squared $\ell _2$-norm of $\varvec{\eta }$. This is accomplished by using $\frac{M\sigma ^2}{\Vert \varvec{A}\varvec{x}_t - \varvec{y}\Vert _2^2}$ as a multiplicative correction term, $c_t$. In each iteration, the value of $s_t^2$ is multiplied with $c_t$ to promote the ratio $\frac{M\sigma ^2}{\Vert \varvec{A}\varvec{x}_t - \varvec{y}\Vert _2^2}$ to be one. The value of $\alpha >0$ (Line 6 in Algorithm 2) controls the contributions of the corrective term, with larger values leading to a more rapid adjustment of $\sigma ^2_t$. Optionally, a user-defined scalar $\tau > 0$ can be used to provide further control over the final strength of the denoiser, with smaller values generating noisy but sharper images. Note, adjusting $s_t^2$ directly is much more challenging as it needs to be varied over iterations, while the fixed value of $\tau$ can be selected once and then kept constant for a given MRI application.

**Algorithm 2:**
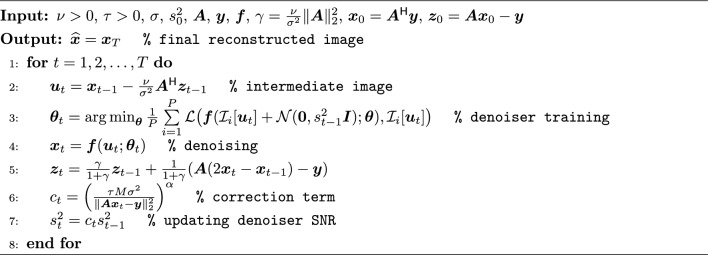
ReSiDe-S

### ReSiDe-M

There are two major limitations of ReSiDe-S. First, it requires de novo training of a denoising network in each iteration, which is time-consuming and thus unrealistic for clinical deployment. Second, ReSiDe-S is a scan-specific method and strictly operates on a single set of undersampled measurements. However, for most MRI applications, more than one set of undersampled measurements is generally available. To reduce computation time at the time of inference and to leverage the availability of multiple sets of undersampled measurements, we propose ReSiDe-M, which is outlined in Algorithm 3. Here, the tilde annotation on the top of the symbol indicates it is a joint representation of $K \ge 1$ sets of measurements. For example, $\tilde{\varvec{A}}$, $\tilde{\varvec{y}}$, and $\tilde{\varvec{\sigma }}^2$, indicate the forward operator, k-space measurements, and average noise variance, respectively, from *K* sets of measurements.

**Fig. 1:**
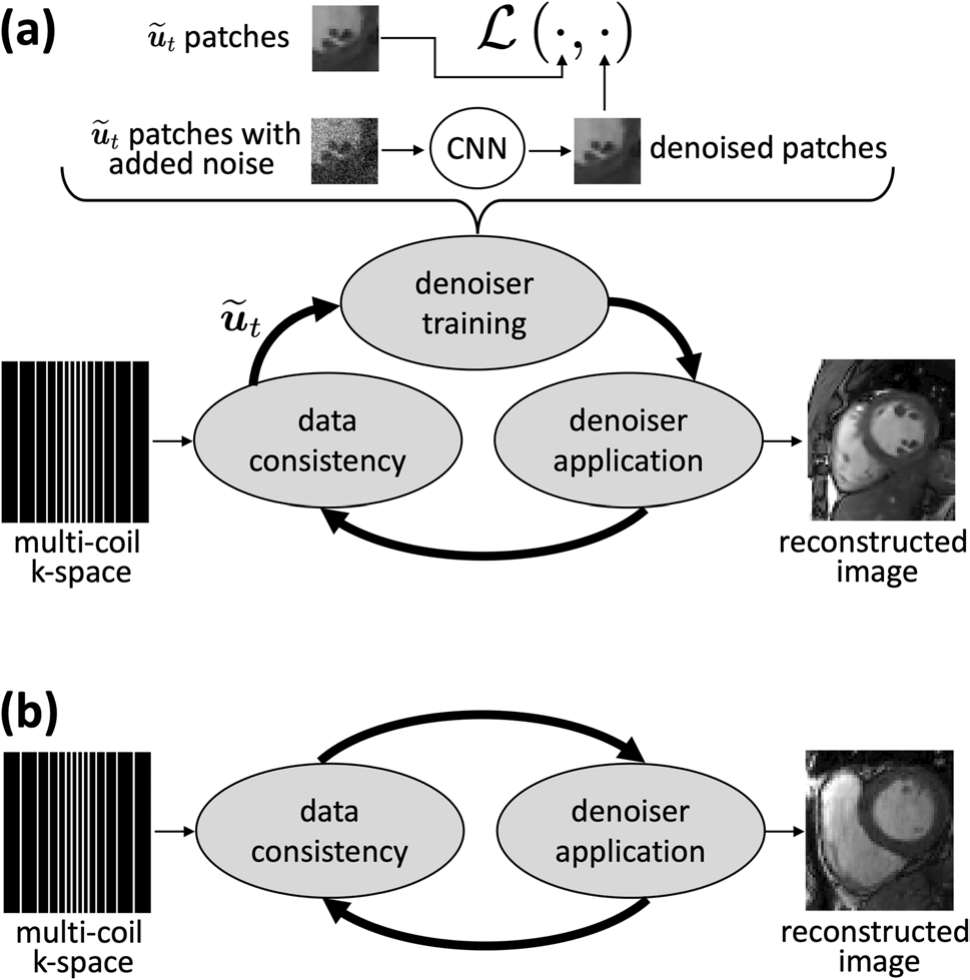
A high-level description of ReSiDe-M. In the training stage (**a**), a convolutional neural network (CNN)-based denoiser is trained on patches from intermediate images $\tilde{\varvec{u}}_t$ (Line 3 in Algorithm 3). The resulting sequence of denoisers is stored. In the inference stage (**b**), the reconstruction is performed using a PnP method, which conceptually alternates between data consistency and denoising steps. The denoising in **b** is performed by calling the denoisers from (**a**), without further training

ReSiDe-M is implemented in two stages: training and inference. The training stage is similar to ReSiDe-S, where both image recovery and denoiser training happen in tandem. However, in contrast to ReSiDe-S, the denoiser training in ReSiDe-M is performed using multiple undersampled datasets. For $K=1$, the training phase of ReSiDe-M is identical to ReSiDe-S. Although the training stage of ReSiDe-M can reconstruct images, its main objective is to store the resulting sequence of denoisers, parameterized by $\{\varvec{\theta }_t\}_{t=1}^T$. The second stage in ReSiDe-M is inference, where an unseen undersampled dataset is reconstructed using a PnP algorithm, with the denoising in the $t^{\text {th}}$ iteration of PnP (Line 11 in Algorithm 3) performed using the denoiser $\varvec{f}(\,\cdot ;\varvec{\theta }_t)$ trained in the first stage. The computational complexity of ReSiDe-M at the inference stage is similar to that of sparsity-based iterative CS methods. A high-level description of ReSiDe-M is given in Fig. 1.

**Algorithm 3:**
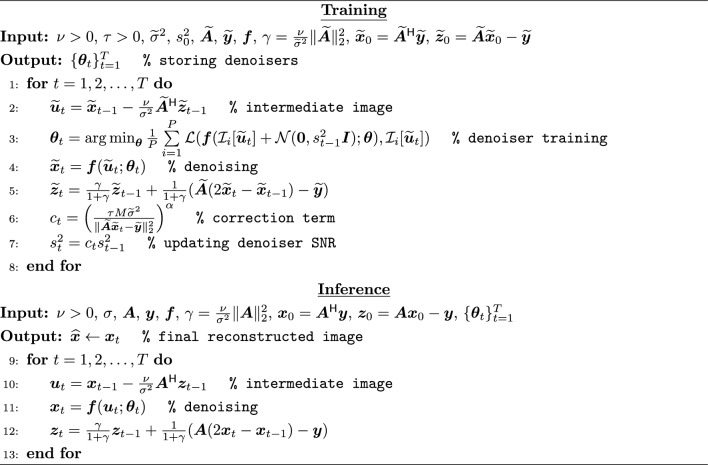
ReSiDe-M

### Study I–brain MRI

In this study, ReSiDe-S and ReSiDe-M were evaluated on T1- and T2-weighted images from the fastMRI dataset [5]. For each contrast, twenty-two sets of multi-coil measurements were used. All T1 images were cropped to $320\times 320$, and all T2 images were cropped to $384\times 304$. Since the noise pre-scan was not available for the fastMRI data, noise pre-whitening was not applied. The multi-coil k-space data were compressed to eight virtual coils [41]. The data were retrospectively downsampled at $R=4$ using two realistic Cartesian sampling masks, i.e., a 1D pseudo-random mask with a 32-line wide ACS region (M1) and a 1D random mask with a 32-line wide ACS region (M2). The M1 mask was kept fixed across training and testing sets of measurements, while a different M2 mask was randomly drawn for each training and testing instance. The coil sensitivity maps were estimated using ESPIRiT [42]. Sixteen out of the 22 sets of measurements were used for network training in ReSiDe-M and SSDU. Five measurement sets were used for performance evaluation and for comparing ReSiDe-S and ReSiDe-M with CS, PnP-BM3D, ConvDecoder [43], and SSDU [25]. The CS reconstruction employed $\ell _1$ regularization in the wavelet domain and was implemented using SigPy [44]. The PnP-BM3D reconstruction was implemented using Algorithm 1, with $\varvec{f}(\cdot )$ in Line 3 of Algorithm 1 representing a call to the BM3D denoiser [45]. To benchmark against a well-established supervised learning method, we further compare ReSiDe-M with a VarNet model pre-trained on brain fastMRI data using rate-4 equispaced (ES) sampling and 8% ACS, resulting in a net acceleration rate of 3.2 [7].

For each method, one set of measurements was used for manual parameter tuning to maximize peak SNR (PSNR). These parameters included: regularization strength, $\lambda$, for CS, denoising level for BM3D in PnP-BM3D, number of iterations and input size for ConvDecoder, cardinality of the loss mask and number of epochs for SSDU, and parameters $\alpha$ and $\tau$, number and the size of patches, and total number of iterations, *T*, for ReSiDe-S and ReSiDe-M. For CS, the reconstruction process was repeated with the regularization set at $\lambda /2$ and $2\lambda$ to assess the impact of regularization strength on the image quality.

### Study II–MRXCAT perfusion phantom

Twenty-two sets of perfusion image series from the MRXCAT digital phantom were considered in this study [46]. Each set of measurements was cropped to $112 \times 168$ pixels, with 32 frames and 4 receive coils. All data were retrospectively downsampled at $R=4$ using a 1D pseudo-random Cartesian sampling mask (M3) [47]. Due to the interleaving nature of M3, the ACS region was not acquired for individual frames, and the fully sampled time-averaged k-space was used to estimate coil sensitivity maps. To simulate realistic data, circularly symmetric zero-mean white Gaussian noise was added to k-space measurements to generate a signal-to-noise ratio of approximately 12 dB. Sixteen sets of measurements were used to train ReSiDe-M, and five sets of measurements were used for performance evaluation and for comparing ReSiDe-S and ReSiDe-M with CS and PnP-BM4D. The CS reconstruction employed $\ell _1$ regularization in the spatiotemporal wavelet domain [48]. The PnP-BM4D reconstruction was implemented using Algorithm 1, with $\varvec{f}(\cdot )$ in Line 3 of Algorithm 1 representing a call to the BM4D denoiser [49]. As described in the previous study, one set of measurements was used to manually optimize free parameters in each method.

### Study III–first-pass perfusion

This study included 22 first-pass perfusion image series from patients clinically referred for a cardiac MRI exam at our institute. All measurements were performed on a commercial 1.5T scanner (MAGNETOM Sola, Siemens Healthcare, Erlangen, Germany) with a fast low angle shot (FLASH) sequence using echo-planar imaging (EPI) readout. The data were collected in three different views, i.e., short-axis, two-chamber, and four-chamber views. The other imaging parameters were: flip angle 25 degrees, temporal footprint 75.48 to 99.36 ms, matrix size $144\times 108$ to $144\times 144$, field of view $360 \times 270$ to $420 \times 380$, echo train length 4, echo spacing 6.06 to 6.29 ms, slice thickness 8 to 10 mm, and a number of frames 60. The images were prospectively undersampled in the $k_x$-$k_y$ domain with an acceleration rate of two and uniform undersampling that was interleaved across time. Noise pre-whitening was applied by using the noise pre-scan, and the multi-coil k-space data were compressed to eight virtual coils [41]. Sixteen sets of measurements were used to train ReSiDe-M, and five sets of measurements were used for performance evaluation and comparison with PnP-BM4D and CS with $\ell _1$ regularization in the spatiotemporal wavelet domain [48]. Again, one set of measurements was used to manually optimize free parameters.

### Quality assessment

In Studies I and II, where the fully sampled reference was available, image quality was assessed using the structural similarity index (SSIM) and PSNR in dB, defined as $20\log _{10}\left( \sqrt{N}|\varvec{x}|_\text {max} / \Vert \varvec{x} - \hat{\varvec{x}}\Vert _2\right)$, with $|\varvec{x}|_{\text {max}}$ being the maximum absolute value in $\varvec{x}$. For Study III, where the fully sampled reference was not available, each perfusion image series was blindly scored by three expert reviewers, including two cardiologists, each with more than ten years of experience in cardiac MRI. Each image series, presented as a movie, was scored on a five-point Likert scale (1: non-diagnostic, 2: poor, 3: adequate, 4: good, 5: excellent) in terms of overall image quality.

### Implementation of ReSiDe

**Fig. 2:**
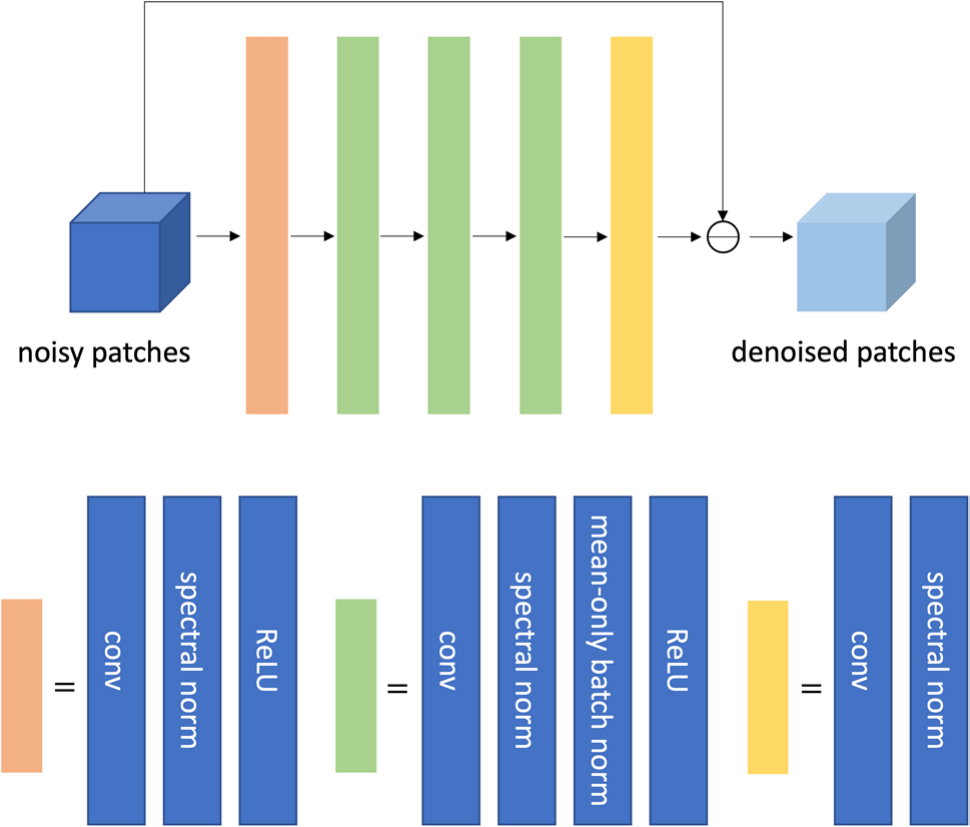
The denoiser architecture used in ReSiDe-S and ReSiDe-M. Here, “conv” represents 2D convolutional layer for Study I and 3D convolutional layer for Studies II and III

In Study I, randomly positioned $P=576$ patches and $P=2,\!306$ patches were used to train the denoiser in ReSiDe-S and ReSiDe-M, respectively. For ReSiDe-M, the $2,\!306$ patches were evenly distributed across the 16 training images. The patch size was fixed at $64 \times 64$. For Studies II and III, randomly positioned $P=288$ patches and $P=4,\!608$ patches were used to train the denoiser in ReSiDe-S and ReSiDe-M, respectively. For ReSiDe-M, the $4,\!608$ patches were again evenly distributed across the 16 training image series. The patch size was fixed at $64 \times 64 \times 20$. In all three studies, the locations of patches were randomly shuffled from one epoch to the next. The mean squared error was used as a cost function to train the denoiser. The real and imaginary parts were split into two channels. The architecture of the denoiser is shown in Fig. 2. Each convolutional layer had 128 kernels of size $3 \times 3$ for Study I and size $3 \times 3 \times 3$ for Studies II and III. We used Adam optimizer with the learning rate $10^{-3}$ for Study I and $10^{-4}$ for Studies II and III. The training in ReSiDe-M was performed on an NVIDIA RTX 2080 Ti for Study I and an NVIDIA RTX 3090 for Studies II and III. For Study I, the measurement noise variance $\sigma ^2$ was approximated from the outer fringes of k-space. For Study II, the noise was synthetically added; therefore, the value of $\sigma ^2$ was precisely known. For Study III, the value of $\sigma ^2$ was inferred from the data scaling factor applied after noise pre-whitening. The value of $\tau$ was set at 0.65, 0.9, and 1.15 for Studies I, II, and III, respectively, and the value of $\alpha$ was set at 0.1 for all studies. The value of $s_0^2$ was set such that the initial SNR for the denoiser training was 5 dB. The number of iterations, *T*, for ReSiDe-S and ReSiDe-M was set at 80 for all three studies. Within each iteration, the denoiser was trained for a total of ten epochs. The code for ReSiDe-S and ReSiDe-M is available here https://github.com/OSU-MR/reside

## Results

### Study I–brain MRI

**Fig. 3:**
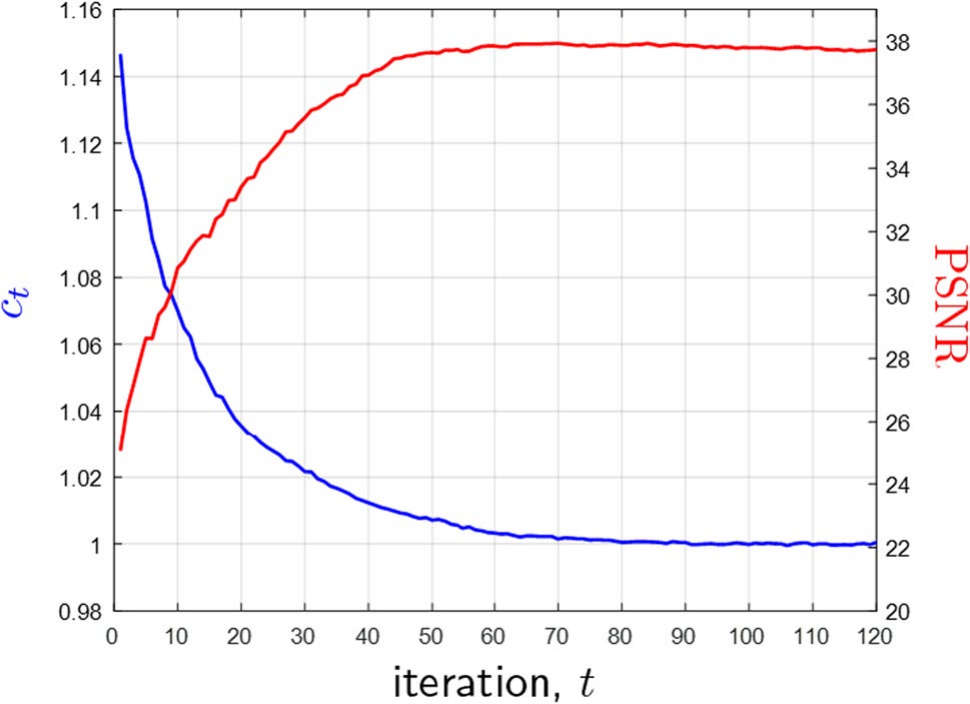
Peak SNR (PSNR) and the multiplicative correction term, $c_t$, as a function of iteration number, *t*. These representative curves originate from one of the T1-weighted images reconstructed using ReSiDe-S

**Fig. 4:**
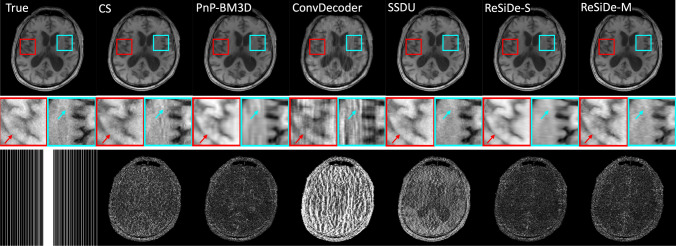
An example showing reconstruction of a T1-weighted image with sampling mask M1. To highlight differences, the second row magnifies two areas in the brain. The arrows point to features where some of the methods show artifacts or blurring. The third row shows the sampling mask (left) and the absolute error maps after five-fold amplification

**Fig. 5:**
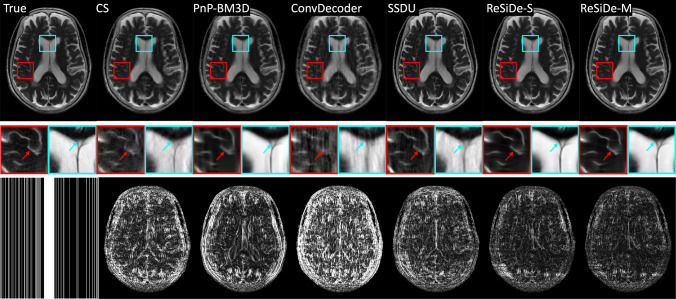
An example showing reconstruction of a T2-weighted image with sampling mask M2. To highlight differences, the second row magnifies two areas in the brain. The arrows point to features where some of the methods show artifacts or blurring. The third row shows the sampling mask (left) and the absolute error maps after five-fold amplification

Figure 3 shows a representative example where the values of PSNR and the multiplicative correction term, $c_t$, are plotted as a function of the number of iterations for ReSiDe-S. Figures 4 and 5 display examples of reconstructed T1- and T2-weighted images using undersampling masks M1 and M2, respectively. The first row presents the true image obtained from fully sampled k-space, alongside the images reconstructed by CS, PnP-BM3D, ConvDecoder, SSDU, ReSiDe-S, and ReSiDe-M. The second row features two magnified regions from the images in the first row. The arrows highlight areas where visible artifacts or blurring is present in some of the reconstructed images. In the third row, the leftmost panel shows the undersampling masks, while the remaining panels depict error maps from various reconstructions after a five-fold amplification. The top section of 1 summarizes PSNR and SSIM values averaged over five T1- and T2-weighted images employing M1 and M2 masks. The PSNR/SSIM of CS reduced from 32.85/0.921 to 32.02/0.907 and 32.40/0.917 when the value of the regularization strength was changed to $\lambda /2$ and $2\lambda$, respectively, indicating that the value of $\lambda$ selected in CS was optimal or near-optimal.

**Fig. 6:**
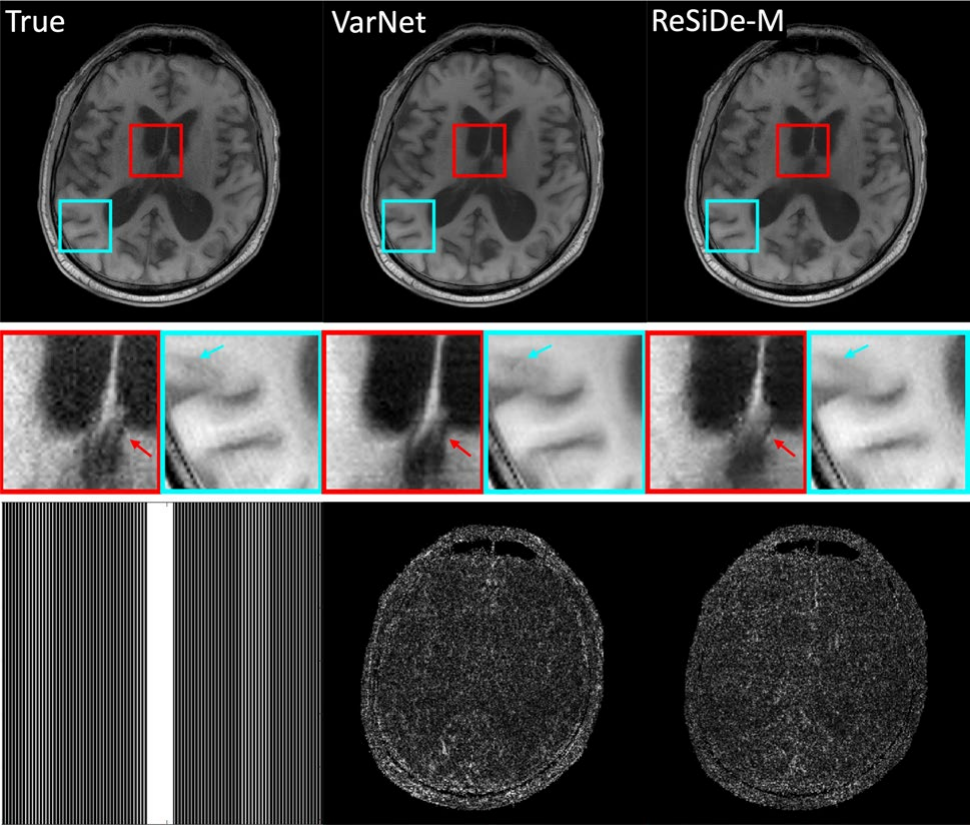
An example showing reconstruction of a T1-weighted image with equispaced (ES) sampling. To highlight differences, the second row magnifies two areas in the brain. The arrows point to features where the two reconstructions show subtle differences. The third row shows the sampling mask (left) and the absolute error maps after five-fold amplification

The comparison between ReSiDe-M and VarNet is summarized in Table 2. Overall, ReSiDe-M compares favorably to VarNet. Despite being trained on the entire fastMRI dataset, the PSNR of VarNet, when averaged over ten T1 and T2 test images, is only 0.27 dB higher than that of ReSiDe-M. Figure 6 shows an example of reconstructed T1-weighted image. Although VarNet reconstruction appears slightly clearer, the differences between the reconstructions are subtle. It is worth mentioning that when the VarNet trained on ES sampling was applied to M1 and M2 sampling patterns, its resulting PSNR value of 32.97 dB was 3.78 dB worse than the value of 36.75 dB achieved by ReSiDe-M. This is because the performance of end-to-end methods, like VarNet, typically degrades when the forward model at the inference stage is different from the one used during training. In contrast, self-supervised methods like ours circumvent this limitation.

**Table 1:** Image quality metrics for Study I (top five rows) and Study II (bottom row)

Image	Samp	CS	PnP-BMXD	ConvDecoder	SSDU	ReSiDe-S	ReSiDe-M
Brain T1	M1	34.55/0.932	35.84/0.949	26.15/0.837	33.28/0.946	35.89/0.947	$\varvec{36.26/0.950}$
Brain T1	M2	30.07/0.874	30.23/0.923	25.63/0.782	31.34/0.925	$\varvec{34.47/0.947}$	34.32/0.946
Brain T2	M1	34.64/0.944	36.54/0.960	27.01/0.841	32.99/0.943	39.06/0.972	$\varvec{39.09/0.973}$
Brain T2	M2	32.13/0.932	33.47/0.940	26.73/0.824	31.35/0.934	$\varvec{37.37/0.976}$	37.34/0.975
Brain Avg	M1/M2	32.85/0.921	34.02/0.943	26.38/0.821	32.24/0.937	36.70/0.961	$\varvec{36.75/0.961}$
MRXCAT	M3	34.91/0.877	36.67/0.900	–	–	38.79/0.918	$\varvec{38.98/0.921}$

In each cell, the first number represents PSNR in dB and the second number represents SSIM, both averaged over five test samples. The best value in each row is highlighted in bold font. The “Brain Avg” row represents the average of the preceding four rows. BMXD represents BM3D for Study I and BM4D for Study II

### Study II–MRXCAT perfusion phantom

**Fig. 7:**
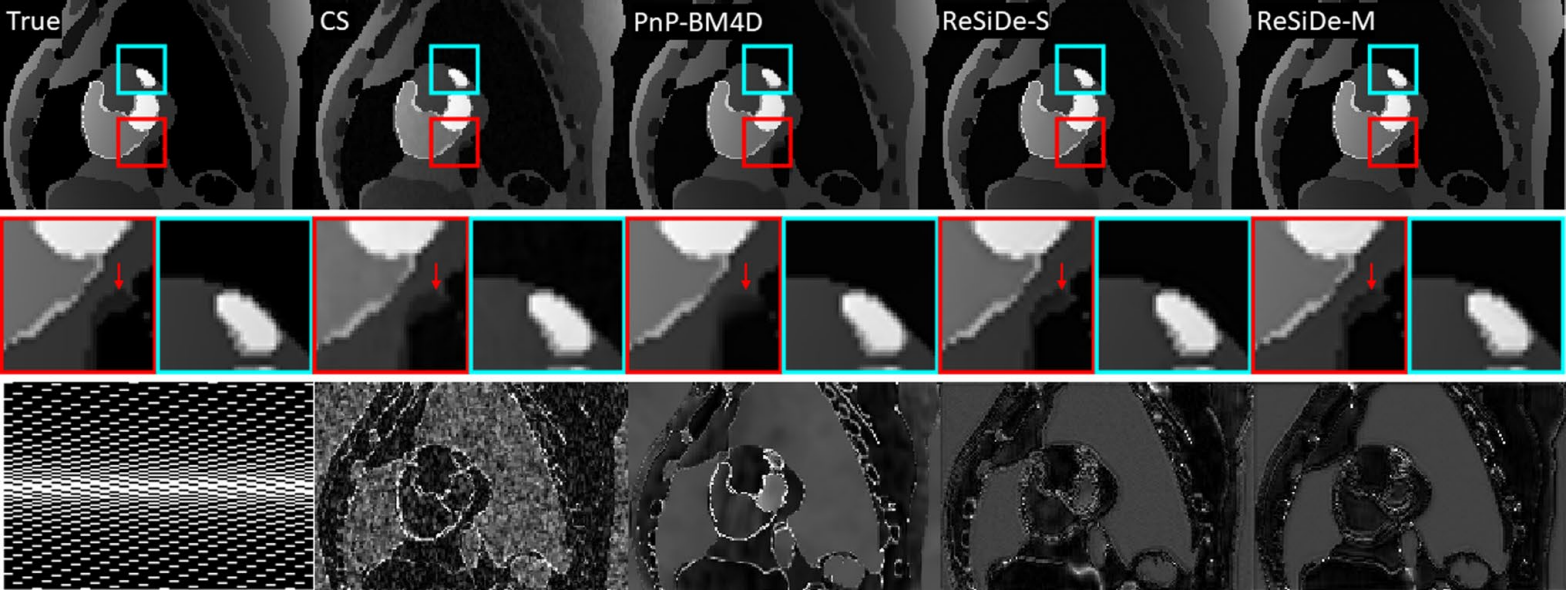
A representative frame from MRXCAT perfusion phantom reconstruction. The second row magnifies two areas of the phantom. The arrows point to features where some of the methods show blurring. The third row shows the sampling mask (left) in the phase-encoding (vertical) and temporal (horizontal) dimensions and the absolute error maps after five-fold amplification

Figure 7 presents a representative frame from reconstructions of an MRXCAT perfusion phantom. The first row displays the true image derived from fully sampled k-space, as well as images reconstructed using CS, PnP-BM4D, ReSiDe-S, and ReSiDe-M methods. The middle row shows a magnification of two selected regions. The arrows emphasize details that are partially or entirely lost in some of the reconstructed images. In the bottom row, the leftmost panel shows the undersampling masks in phase encoding (vertical) and temporal (horizontal) dimensions. The readout dimension, which is not shown, is fully sampled. The remaining panels in the third row depict error maps after a five-fold amplification. The last row in Table 1 summarizes PSNR and SSIM values averaged over five MRXCAT datasets with M3 mask for CS, PnP-BM4D, ReSiDe-S, and ReSiDe-M.

**Table 2:** Comparison of ReSiDe-M and VarNet for equispaced (ES) sampling

Image	Samp	VarNet	ReSiDe-M
Brain T1	ES	36.02/0.961	$\varvec{37.28/0.962}$
Brain T2	ES	$\varvec{40.98/0.985}$	39.17/0.973
Brain Avg	ES	$\varvec{38.50/0.973}$	38.23/0.968

**Table 3:** Image quality scores from three expert reviewers (E1, E2, and E3) on a five-point Likert scale (5: best, 1: worst), averaged over five perfusion image series

	CS	PnP-BM4D	ReSiDe-S	ReSiDe-M
E1	3.2	4.0	4.4	$\mathbf {5.0}$
E2	2.0	3.0	4.0	$\mathbf {4.6}$
E3	2.0	3.4	3.6	$\mathbf {3.6}$
Avg	2.4	3.5	4.0	$\mathbf {4.4}$

### Study III–first-pass perfusion

**Fig. 8:**
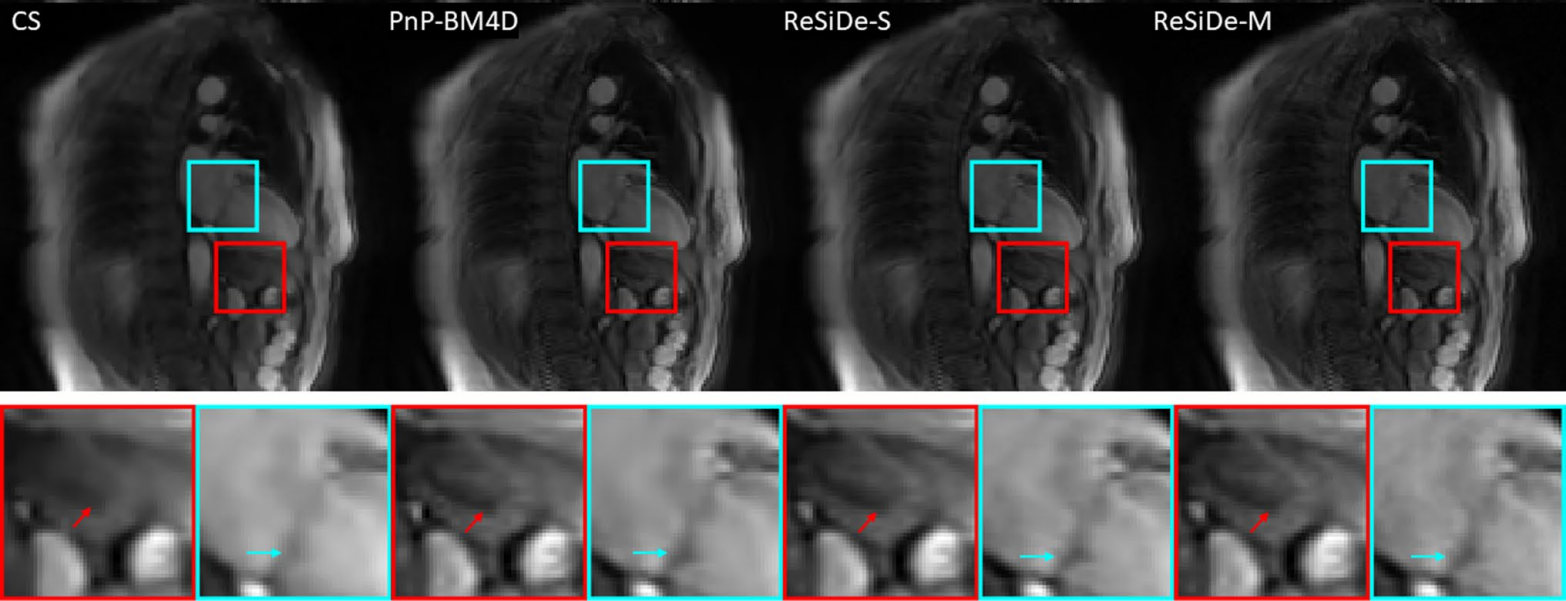
A representative two-chamber frame from one of the first-pass perfusion image series. The first row shows the entire frame, while the second shows two magnified areas from the frame. The visible loss of detail in CS is highlighted with arrows

**Fig. 9:**
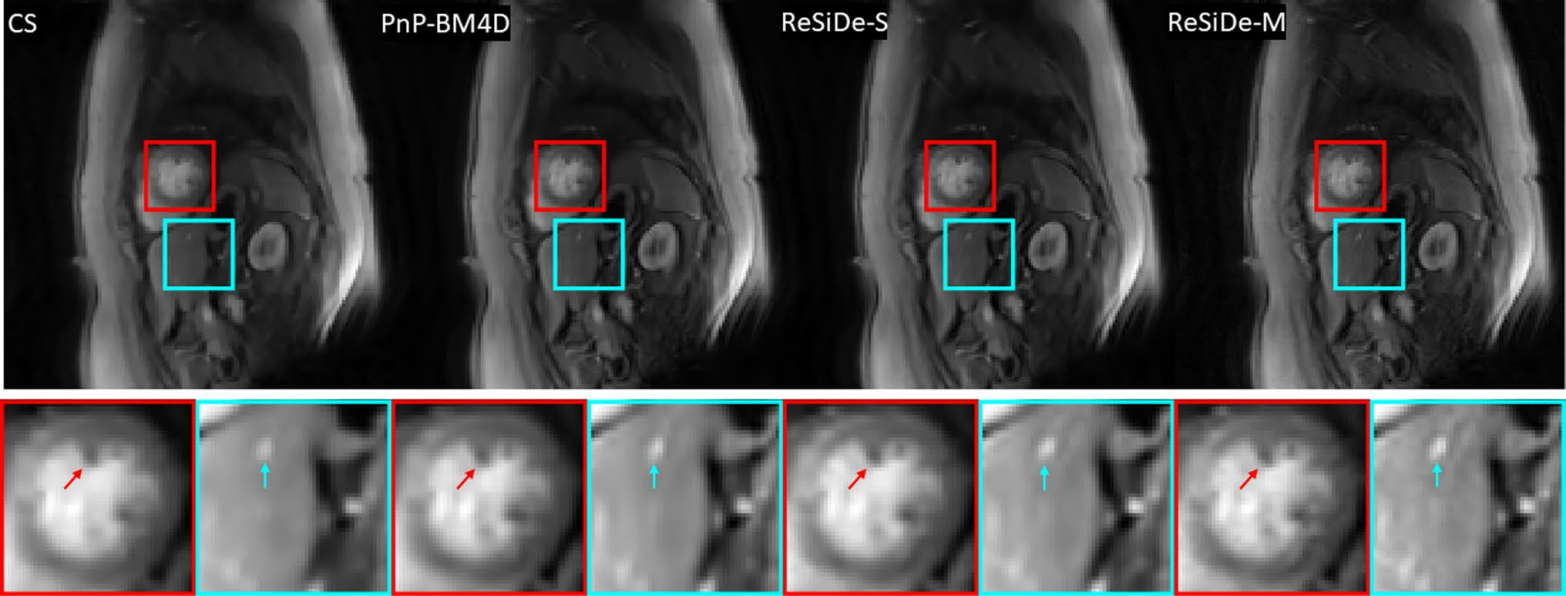
A representative short-axis frame from another first-pass perfusion image series. The first row shows the entire frame, while the second shows two magnified areas from the frame. The visible loss of detail in CS is highlighted with arrows

Figures 8 and 9 show representative frames from two different first-pass perfusion image series. Reconstructions from CS, PnP-BM4D, ReSiDe-S, and ReSiDe-M are shown. The top row shows the entire frame, and the bottom row displays two magnified regions from the images in the first row. The arrows highlight the details that are partially lost in some of the reconstructed images. In Fig. 8, the cyan arrows point to the leaflets of the mitral valve. In Fig. 9, the red arrows point to the papillary muscles in the left ventricle. For CS, PnP-BM4D, ReSiDe-S, and ReSiDe-M, Table 3 provide the image quality scores averaged over five image series from three cardiac MRI experts, including two cardiologists.

## Discussion

In this work, we present an SSDL method, called ReSiDe, for MRI reconstruction. Like PnP methods, ReSiDe integrates a denoiser into the reconstruction process. However, PnP methods use pre-trained denoisers, while ReSiDe iteratively trains the denoiser on the images being recovered. We present two variations of ReSiDe, i.e., ReSiDe-S and ReSiDe-M. ReSiDe-S is scan-specific and utilizes only a single set of undersampled measurements. The necessity to train the network in each iteration makes ReSiDe-S computationally slow. In contrast, ReSiDe-M operates on multiple sets of undersampled measurements and thus employs a more comprehensive training of the denoiser using patches from multiple images. The denoisers trained in ReSiDe-M are stored and then utilized in a PnP algorithm without further training. The computation burden of ReSiDe-M after the training stage is comparable to CS-based iterative methods.

Validation of ReSiDe-S and ReSiDe-M is carried out in three datasets, i.e., T1- and T2-weighted images from fastMRI (Study I), digital perfusion image series from MRXCAT (Study II), and first-pass perfusion data collected from patients (Study III). In Study I, we compare ReSiDe-S and ReSiDe-M with other methods that do not require fully sampled data, including CS, PnP-BM3D, ConvDecoder, and SSDU. As summarized in 1, ReSiDe-M and ReSiDe-S outperform competing methods in terms of PSNR and SSIM, with more than 2.5 dB improvement over PnP-BM3D and SSDU. All methods perform better with the M1 mask compared to the M2 mask. This is not surprising because M1 is pseudo-random and thus avoids large sampling gaps in k-space. Also, M1 is kept consistent between training and testing, while a different M2 mask is used for each training and testing instance. Nonetheless, the smaller performance gap between M1 and M2 for ReSiDe highlights its ability to generalize when the sampling patterns between training and testing are different. Two examples of reconstructed images are shown in Figs. 4 and  5. Compared to PnP-BM3D, ConvDecoder, and SSDU, both ReSiDe-S and ReSiDe-M exhibit fewer artifacts while preserving fine details. The artifacts are particularly pronounced in ConvDecoder. Despite our best efforts to optimize the code provided by the original authors here [50], we were unable to improve the performance of ConvDecoder. The performance of CS was inferior to ReSiDe-S and ReSiDe-M by more than 3 dB. To explore if this performance gap is due to the suboptimal selection of the regularization strength in CS, we repeated the CS reconstruction at lower ($\lambda /2$) and higher ($2\lambda$) regularization strengths. However, the performance of CS degraded for both those choices, indicating that the inferior performance of CS cannot be attributed to suboptimal selection of $\lambda$.

The PSNR and SSIM numbers from Study II followed a trend similar to that of Study I, with both ReSiDe-S and ReSiDe-M outperforming other methods. Figure 7 shows a representative frame. As shown in the error map, ReSiDe-S and ReSiDe-M preserve edge information more effectively, whereas CS and PnP-BM4D show structured errors around the edges. The performance gap is more than 1.5 dB between ReSiDe and PnP-BM4D and more than 3 dB between ReSiDe and CS.

In Study III, where the image series were subjectively evaluated by three expert readers, ReSiDe-M consistently outperformed other methods, with ReSiDe-S being the second best. The example images provided in Figs. 8 and 9 illustrate that the ReSiDe methods are more effective in preserving small details, e.g., mitral valve leaflets or papillary muscles, especially compared to CS.

The performance difference between ReSiDe-S and ReSiDe-M, in terms of PSNR and SSIM, was marginal in Studies I and II. However, we observed that ReSiDe-M images have more texture and appear sharper than those from ReSiDe-S. We conjecture that this behavior is related to more expansive training of the denoiser in ReSiDe-M. In addition to the superior image quality, ReSiDe-M also offers a computation advantage over ReSiDe-S and many other SSDL methods. For example, in Study I, ReSiDe-S and ReSiDe-M trainings took 36 min and 140 min, respectively. However, at the inference stage, the reconstruction from ReSiDe-M took only 11 s per image. In comparison, the training and inference from SSDU took 127 min and 3.3 s, respectively. The inference time for ReSiDe-M for Studies II and III was 25 and 37 s, respectively, per image series. In comparison, PnP-BM4D took 40 min and 110 min for each image series in Studies II and III, respectively.

The performance of ReSiDe depends on $s_t^2$, which controls the training SNR of the denoiser. We employ the discrepancy principle to adapt the value of $s_t^2$. Figure 3 shows representative curves for PSNR and $c_t$ for one the T1-weighted images. As expected, the value of the corrective term, $c_t$, converges to one after approximately 80 iterations, which implies that $s_t^2$ also converges to a fixed value. Likewise, the PSNR reaches its maximum value at around the 60 iteration mark, with marginal or no drop-off in PSNR for iterations beyond 60. Similar trends were observed for other datasets in Studies I, II, and III. The value of $\alpha$ had a marginal impact on the final PSNR but it did affect the rate of convergence. In particular, larger values of $\alpha$ led to faster convergence but with PSNR and $c_t$ curves showing more oscillations, especially in the earlier iterations. Therefore, the value of $\alpha$ was conservatively set at 0.1. Also, the performance of ReSiDe does not vary significantly with the number of patches as long as that number is large enough. The values of $\tau$ and patch size do affect image quality and were optimized on a separate set of measurements in each study.

This study has several limitations. First, our current implementation requires saving a denoiser in each iteration of the training process. Saving a large number of denoisers can be memory intensive, especially if larger networks are employed. Future work could explore saving the denoisers less frequently, e.g., after every tenth iteration. Second, we have used a denoiser architecture that is based on the residual learning approach proposed in 2017 [51]. It is possible that more recent network architectures that utilize attention mechanisms [52] can further improve the performance of ReSiDe. Third, the denoiser training used in ReSiDe does not explicitly use the g-factor information, and using this information can further improve the performance [53]. Fourth, although using the discrepancy principle eliminates the need to precisely schedule the denoiser strength [32], both ReSiDe-S and ReSiDe-M still require selecting an appropriate value for $\tau$. For the studies presented, we manually selected one value of $\tau$ for each application. It is not clear whether this value of $\tau$ will remain reasonable if imaging parameters, e.g., spatial resolution or measurement SNR, change significantly within each application. However, ReSiDe shares this limitation with CS and PnP methods, which also require selecting the regularization or denoising strength. Fourth, we observed ReSiDe-S and ReSiDe-M to converge to a fixed point in all three represented studies. However, a more formal convergence analysis for ReSiDe is currently missing and beyond the scope of this proof-of-concept work.

## Conclusions

We have presented two self-supervised methods for MRI reconstruction: ReSiDe-S and ReSiDe-M. ReSiDe-S offers a scan-specific implementation where a single set of undersampled measurements is used for denoiser training and image recovery. In contrast, ReSiDe-M trains a denoiser from multiple undersampled measurements and utilizes that denoiser during inference without further training. Our validation studies, which used data from brain MRI, perfusion phantom, and first-pass perfusion, demonstrate that ReSiDe-S and ReSiDe-M outperform other self-supervised or unsupervised methods in terms of both qualitative and quantitative metrics. Compared to ReSiDe-S, ReSiDe-M also offers slightly better image quality and much faster inference.

## Data Availability

The code and data for ReSiDe-S and ReSiDe-M can downloaded from here https://github.com/OSU-MR/reside.
